# A Three–Dimensional Ontological Formulation of Bohmian Mechanics

**DOI:** 10.3390/e28040382

**Published:** 2026-03-31

**Authors:** Pablo P. Yepes

**Affiliations:** Physics and Astronomy Department, Rice University, Houston, TX 77005, USA; yepes@rice.edu

**Keywords:** quantum mechanics, ontology, 3D, Bohmian mechanics, pilot-wave, de Broglie–Bohm, trajectories, 3D wave

## Abstract

We present a three–dimensional field ontology for Bohmian mechanics in which the single-particle wave functions defined in ordinary physical space are taken as ontological elements alongside particle positions. In this view, each particle is associated with a wave function in three–dimensional space conditioned on the actual positions of the remaining particles. The universal wave function on configuration space is retained within the formalism, but its role is interpreted as nomological: it determines the coupled evolution of the three–dimensional fields without itself being treated as a fundamental physical field. The corresponding evolution equations for the conditioned three–dimensional wave functions follow from the standard Bohmian formalism and, together with the guidance equation, yield dynamics fully equivalent to those of the conventional theory. The empirical and predictive content of Bohmian mechanics is thereby preserved, while its ontological structure is expressed in explicitly three–dimensional terms. Within a ψ-ontic perspective, this proposal makes explicit an ontological organization of Bohmian mechanics in which the dynamical fields are defined on physical space. While fully equivalent to the conventional formulation, expressing the theory in these terms may facilitate interpretation, visualization, and pedagogical presentation.

## 1. Introduction

The ontology of quantum mechanics remains one of its most contested features. Bohmian mechanics offers a realist alternative to standard interpretations by positing particles with definite positions guided by a universal wave function [1,2]. However, this wave function, $\Psi (x_{1},\ldots ,x_{N},t)$, is a field on the high–dimensional configuration space $R^{3N}$, not on the three–dimensional physical space we inhabit. This raises a central interpretative challenge: How much are we to read our mathematical ‘maps’ as a literal description of the physical ‘territory’? The fact that alternative, empirically equivalent formulations exist—such as the Heisenberg picture, which suggests a different ontology of evolving operators rather than evolving states—underscores that a given formalism is not a given reality.

This paper represents a realist project that adopts the Schrödinger picture as the most intuitive foundation for an ontology of beables evolving in time. From this starting point, we suggest that the standard interpretation, by reifying configuration space, conflates the mathematical ‘map’ with the physical territory. In contrast, we propose a reinterpretation of Bohmian mechanics in which single-particle wave functions—traditionally treated as technical derivatives and referred to as “conditional wave functions”—are elevated to *ontological primitives*, referred to here as 3D wave functions.

In this framework, each particle is associated with a wave function in real three–dimensional space, conditioned on the actual positions of the others. Consequently, the guiding equation is naturally expressed in 3D space, while the full dynamical content of the theory is preserved. It is important to emphasize that this proposal does not yield new empirical predictions; the formal dynamics remain those of standard Bohmian mechanics. The novelty lies instead in the ontological shift: by relocating the wave function from configuration space to a family of interacting 3D fields, we achieve conceptual clarity and pedagogical accessibility.

Recent no-go results in the foundations of quantum mechanics sharpen the ontological stakes of these questions. In particular, the Pusey–Barrett–Rudolph (PBR) theorem rules out a broad class of epistemic interpretations of the quantum state [3], supporting the conclusion that the wave function corresponds to physical reality rather than mere information. Importantly, however, the PBR theorem is neutral with respect to the spatial representation of that reality: it constrains the ontological status of the quantum state, but not the space in which it is realized. Configuration-space realism is therefore one possible response to ψ-ontology, but it is not forced by it. The present work explores an alternative resolution of this underdetermination in which the ontic content required by PBR is carried by wave-like fields defined directly in three–dimensional space. By elevating the conditional (or “3D”) wave functions of Bohmian mechanics to ontological primitives, we propose that a ψ-ontology can be maintained without committing to configuration space as the fundamental arena of physical reality.

In what follows, we use the term *ontological primitives* to denote those entities posited as elements of physical reality (beables) that are not reducible to other dynamical variables within the theory. In the present framework, these primitives consist of particle positions together with the associated single-particle wave functions defined in ordinary three–dimensional space.

## 2. Standard Bohmian Formalism

Bohmian mechanics describes an *N*-particle quantum system using the following:

- A universal wave function $\Psi (x_{1},\ldots ,x_{N},t)\in C$, defined on configuration space $R^{3N}$.
- A set of particle positions $x_{i}\left(t\right)\in R^{3}$, evolving according to the guiding equation
  (1) dxidt=vi(t)=ℏmiIm∇xiΨ(x1,…,xN,t)Ψ(x1,…,xN,t)|xj=xj(t).
- Schrödinger evolution of Ψ:
  (2) $$i\hbar \frac{\partial \Psi }{\partial t}=\hat{H}\Psi .$$

This formalism is deterministic, nonlocal, and empirically equivalent to standard quantum mechanics [4]. Yet its reliance on configuration space as the domain of the wave function has motivated various proposals for reformulation or reinterpretation [5,6]. While it is standard to say that the universal wave function $\Psi (x_{1},\ldots ,x_{N},t)$ “lives” on the 3N-dimensional configuration space $R^{3N}$, this is not a necessary view. An equivalent reinterpretation associates with each particle its own wave function in 3D space, with these wave functions interacting through their dependence on the actual positions of the others. Developing this alternative perspective is the aim of the present paper.

## 3. The 3D Wave Function

We now derive the exact evolution equation for the 3D wave function, demonstrating how the familiar single-particle Schrödinger equation is modified by a non-local quantum potential. This derivation is not an approximation but an exact reformulation of the standard Bohmian dynamics.

### 3.1. Defining the 3D Wave Function

We begin with the universal wave function $\Psi (x_{1},\ldots ,x_{N},t)$ on configuration space $R^{3N}$ obeying the standard Schrödinger equation, $i\hbar \partial_{t}\Psi =\hat{H}\Psi$. From the Bohmian trajectories $X_{j}\left(t\right)$ for each particle, we define the 3D wave function for particle *i* by evaluating the configuration-space wave function at the actual positions of all particles except *i*, leaving the coordinate of particle *i* as the only spatial argument:

(3) $$\psi_{i}\left(x,t\right):=\Psi \left(X_{1}\left(t\right),\ldots ,X_{i-1}\left(t\right),\;x,\;X_{i+1}\left(t\right),\ldots ,X_{N}\left(t\right),t\right).$$

This object, typically called the *conditional wave function* [4], is a genuine field on physical three–dimensional space. For our ontological proposal, we elevate its status from a mere technical construct to a primitive beable, referring to it simply as the *3D wave function*.

### 3.2. The Evolution Equation and the Quantum Potential

The evolution of $\psi_{i}\left(x,t\right)$ is determined by the universal Schrödinger equation. To reveal the physical nature of the interactions between the 3D wave functions, we employ a derivation that makes the coupling terms explicit, following the detailed formalism presented in authoritative texts on the subject [7,8]. The evolution law for $\psi_{i}$ can be written as

(4) $$i\hbar \frac{\partial \psi_{i}}{\partial t}=\left(-\frac{\hbar^{2}}{2m_{i}}\nabla_{x}^{2}+V_{i}\right)\psi_{i}+K_{i}\left(x,t\right)\;\psi_{i},$$

where $K_{i}\left(x,t\right)$ is a complex potential that couples particle *i* to all other particles in the system. By performing a polar decomposition of the universal wave function ($\Psi =Re^{iS/\hbar }$) and applying the Bohmian guiding law, this potential can be separated into three distinct parts:

(5) $$K_{i}\left(x,t\right)=\underset{Q_{-i}\left(x,t\right)}{\underset{︸}{\sum_{j\neq i}Q_{j}\left(x,t\right)}}-\underset{T(t)}{\underset{︸}{\sum_{j\neq i}\frac{1}{2}m_{j}{\left|v_{j}\left(t\right)\right|}^{2}}}-i\underset{D(t)}{\underset{︸}{\frac{\hbar }{2}\sum_{j\neq i}\nabla_{x_{j}}\cdot v_{j}\left(t\right)}}.$$

Here, $Q_{j}\left(x,t\right)={\left[-\frac{\hbar^{2}}{2m_{j}}\frac{\nabla_{x_{j}}^{2}R}{R}\right]}_{x_{-i}=X_{-i}\left(t\right)}$ is the quantum potential associated with particle *j*. The crucial insight is that the coupling consists of a position-dependent quantum potential, $Q_{-i}\left(x,t\right)$, and two terms, T(t) and D(t), that depend only on time. These time-dependent terms can be absorbed into a physically irrelevant phase factor via a gauge transformation, $\psi_{i}\left(x,t\right)=e^{\Lambda (t)}\Phi_{i}\left(x,t\right)$. This yields the final, tidy evolution equation for the rescaled 3D wave function $\Phi_{i}$:

(6) $$i\hbar \frac{\partial \Phi_{i}}{\partial t}=\left(-\frac{\hbar^{2}}{2m_{i}}\nabla_{x}^{2}+V_{i}\left(x,X_{-i}\left(t\right)\right)\right)\Phi_{i}+Q_{-i}\left(x,t\right)\;\Phi_{i}.$$

Equation (6) expresses the evolution of the three–dimensional wave functions in terms of local kinetic and potential contributions together with a nonlocal coupling term $Q_{-i}\left(x,t\right)$. The latter encodes the influence of the remaining particles through the universal wave function evaluated at their actual configuration. The derivation of Equations (4)–(6) from standard Bohmian mechanics is presented in Appendix A.

In this way, $\Phi_{i}\left(x,t\right)$ provides a three–dimensional representation of the dynamics in which interparticle correlations are mediated through the coupling structure generated by Ψ. The equation is therefore not autonomous in the absence of Ψ; rather, it reorganizes the standard Bohmian dynamics by making explicit how the configuration-space structure induces effective wave fields defined on physical space.

This lack of autonomy should not be taken to undermine the ontological status of the particle-indexed fields. The $\Phi_{i}$ are not mathematically independent of Ψ and the particle configuration, but this does not preclude assigning them ontological significance. From this perspective, the conditional wave functions represent the physically realized guiding fields associated with each particle, while the universal wave function remains part of the global dynamical structure that fixes their evolution. The proposal is therefore not a reduction of the formalism but a reallocation of ontological roles: from a configuration-space object to a set of interacting fields defined in ordinary three–dimensional space. This reflects a general methodological point: the mathematical state space of a theory need not be identified with the space in which its ontology is realized.

### 3.3. Guidance, Equivariance, and the Axiomatic View

The particle’s motion is guided by its associated 3D wave function. Since the gauge transformation is spatially uniform, the guiding law can be expressed equivalently using $\psi_{i}$ or $\Phi_{i}$:

(7) X˙i(t)=ℏmiIm∇xΦi(x,t)Φi(x,t)x=Xi(t)=1mi∇xSi(x,t)|x=Xi(t).

The non-local term $Q_{-i}\left(x,t\right)$ in the evolution Equation (6) is precisely what is needed to ensure that the dynamics of the 3D fields and particles preserves the quantum equilibrium distribution ${\left|\Psi \right|}^{2}$, a property known as equivariance.

This naturally leads to an alternative, but fully equivalent, axiomatic formulation of Bohmian mechanics:

1. **Ontology:** The ontological primitives consist of particles with positions $X_{i}\left(t\right)\in R^{3}$, together with a set of corresponding fields $\left\{\Phi_{i}\left(x,t\right)\right\}$ on $R^{3}$.
2. **Guidance Postulate:** Each particle is guided by its field via Equation (7).
3. **Evolution Postulate:** The fields evolve according to the coupled evolution Equation (6), where the non-local coupling is given by the quantum potential $Q_{-i}$.

This axiomatic presentation makes the conceptual advantage of the 3D formalism explicit: the fundamental dynamics occur in ordinary 3D space, with non-locality manifesting as an explicit potential in the fields’ evolution equations.

## 4. Conceptual Features of the 3D Ontology

The 3D wave function formalism, while empirically equivalent to the standard model, offers a distinct physical and philosophical perspective. To sharpen its conceptual profile, we emphasize a **two-level ontology**:

- **Ontological Primitives:** The manifest beables are particles with definite positions in 3D space, together with their associated 3D wave functions $\psi_{i}\left(x,t\right)$. These fields are ontological primitives, guiding particle motion via Equation (7).
- **Nomological Structure:** The universal wave function Ψ on configuration space is retained, but only as a compact generator of nonlocal quantum potentials $Q_{-i}\left(x,t\right)$. In this sense, “nomological” means that Ψ enters as part of the dynamical structure governing the evolution of the beables, rather than as a localized physical field defined on space. It encodes the global dynamical constraints without being itself a material field.

This hierarchy clarifies the metaphysical stance: reality consists of particles and their guiding 3D fields, while the universal wave function plays a law-like role analogous to the Hamiltonian in classical mechanics.

### 4.1. Born Rule and Quantum Equilibrium

Since the theme of the present Special Issue concerns Born’s rule, it is important to emphasize explicitly that the proposed 3D ontology preserves the full statistical structure of standard Bohmian mechanics. In Bohmian mechanics, the Born rule arises through the quantum equilibrium hypothesis: for an ensemble of systems with universal wave function $\Psi (x_{1},\ldots ,x_{N},t)$, the distribution of particle configurations is given by

$$\rho \left(x_{1},\ldots ,x_{N},t\right)={\left|\Psi \left(x_{1},\ldots ,x_{N},t\right)\right|}^{2}$$

and this distribution is preserved under the dynamics (equivariance). The present re-expression retains this structure exactly.

When expressed in terms of 3D wave functions, the equilibrium distribution on configuration space induces a corresponding statistical measure over particle positions together with their associated conditional wave functions $\psi_{i}\left(x,t\right)$. In this sense, Born’s rule may be understood as a statistical law governing ensembles of particles and 3D fields rather than as a primitive postulate about configuration space amplitudes. The 3D ontology thus reproduces all quantum statistics while rendering their physical basis more transparent: probabilities reflect equilibrium properties of spatially grounded beables evolving under deterministic, nonlocal dynamics.

Accordingly, the Born rule is neither weakened nor reinterpreted in a way that alters its empirical content. Its role is clarified rather than revised, appearing as a natural consequence of quantum equilibrium within a fully three–dimensional ontology.

### 4.2. Collapse and Conditionalization

In standard Bohmian mechanics, collapse is not a fundamental physical process but an effective one, occurring at the level of subsystem conditional wave functions once environmental degrees of freedom are taken into account. The present framework does not modify the dynamics or empirical content of the theory; it differs only in ontology. The same conditional wave functions that already appear in standard Bohmian mechanics are here treated as ontological primitives in physical space, so that effective collapse is understood as the conditional updating of real-space fields upon the actual configuration of the environment. Expressed in this way, collapse involves no departure from unitary dynamics and no appeal to measurement postulates or consciousness, but becomes a transparent feature of the real-space description.

### 4.3. Physical Transparency vs. Mathematical Compactness

One might note that the *N* coupled equations of motion for the 3D wave functions (Equation (6)) are more complex than the single Schrödinger equation on configuration space. This reflects a deliberate choice: prioritizing physical transparency over mathematical compactness. The additional term $Q_{-i}\left(x,t\right)$ is not mere mathematical clutter, but the explicit representation of non-local quantum interaction in ordinary three–dimensional space. In contrast, the configuration-space formulation provides a compact global encoding of the same dynamics. The two descriptions are mathematically equivalent, but emphasize different aspects of the theory.

### 4.4. Structural Summary

The theory can be summarized in the following structural form: (1) Ontology: A set of particle positions $\left\{X_{i}\left(t\right)\right\}$ together with associated three–dimensional wave functions $\left\{\Phi_{i}\left(x,t\right)\right\}$ defined on physical space. (2) Guidance: Each particle position evolves according to the standard Bohmian guidance equation,

X˙i(t)=ℏmiIm∇ΦiΦix=Xi(t).

(3) Field evolution: Each $\Phi_{i}\left(x,t\right)$ satisfies Equation (6), in which the coupling term $Q_{-i}\left(x,t\right)$ is fixed by the universal wave function Ψ through the standard Bohmian formalism. In this presentation, Ψ is not treated as an additional material field in configuration space but as part of the dynamical structure that determines the coupling among the three–dimensional wave functions.

The present proposal does not eliminate the universal wave function from the formalism. Its contribution is instead to reorganize the ontology of Bohmian mechanics by treating the three–dimensional wave functions as the beables while assigning Ψ a nomological role. What is achieved is therefore not a reduction of mathematical structure but a clarification of ontological commitments within an exactly equivalent dynamical framework.

## 5. Philosophical Positioning and Relation to Alternative Approaches

Having established the dynamics and conceptual features of the 3D ontology, we now position it with respect to other interpretations, including configuration-space realism, the nomological view, and other approaches that seek to ground quantum mechanics in 3D space.

### 5.1. The Choice of Formalism: Realism vs. Non-Realism

Any realist interpretation of quantum mechanics is implicitly rooted in the **Schrödinger picture**, where reality is composed of ‘beables’ (like particles and fields) that have a state and evolve in time. This choice is deliberate.

The primary alternative, the **Heisenberg picture**, is more naturally aligned with the non-realist Copenhagen interpretation. In the Heisenberg picture, the state vector is static, and the ‘observables’ (represented by operators) evolve. This formalism is philosophically suited to an instrumentalist view, where physics is only about the *outcomes of measurements* (observables) and not an underlying ‘story’ of reality. The concept of a ‘beable’ does not naturally arise, if at all.

Our framework, in contrast, is fundamentally realist. By choosing the Schrödinger picture, we accept that there *is* a ‘story’ of reality evolving in spacetime. The central question for us is not *if* there are beables, but *what* they are. The standard Bohmian answer is ‘particles and a global Ψ’. This paper argues for a more coherent answer: ‘particles and their 3D fields $\psi_{i}$’.

Having established our realist foundations in the Schrödinger picture, we now position our 3D ontology with respect to other realist approaches.

### 5.2. Configuration-Space Realism and the 3D Ontology

Proponents of configuration-space realism argue that the universal wave function Ψ represents a genuine physical field on the high-dimensional space $R^{3N}$ [9,10]. On this view, the fundamental ontology of quantum theory is not the familiar three–dimensional world but a vast configuration space in which the wave function “lives,” with ordinary 3D reality treated as emergent or derivative. While this interpretation has the virtue of literalism, it is often argued to incur a substantial metaphysical cost: it reifies an abstract mathematical representation and requires a radical reconceptualization of physical space as non-fundamental.

The present framework offers an alternative that preserves the full empirical and dynamical content of Bohmian mechanics without this ontological commitment. The same dynamics encoded in the configuration-space Schrödinger equation can be expressed exactly in terms of interacting wave functions defined on ordinary three–dimensional space. Each particle is associated with a 3D wave function evolving according to Equation (6), with nonlocal correlations encoded explicitly through the quantum potential $Q_{-i}\left(x,t\right)$. In this formulation, configuration space remains a powerful mathematical tool, but it is no longer interpreted as a physically real arena.

This perspective engages with ongoing debates concerning the ontological status of the configuration-space wave function in Bohmian mechanics. Various authors have questioned whether taking configuration space as fundamental introduces additional metaphysical commitments or explanatory challenges regarding the emergence of the three–dimensional world [6,11]. By relocating the ontology into ordinary space—particles together with their guiding 3D wave functions—the present framework offers an alternative to this line of concern. The theory is formulated entirely in terms of entities in physical space while remaining exactly equivalent to standard Bohmian mechanics at the level of dynamics and empirical content.

In short, the mathematical use of configuration space does not force an ontological commitment to configuration-space realism. The same physics can be represented through a spatially grounded ontology of particles and interacting 3D wave functions.

## 6. PBR, ψ–Ontology, and Conditional Wave Functions

Recent no-go results sharpen the ontological stakes of interpretations of the quantum state. In particular, the Pusey–Barrett–Rudolph (PBR) theorem shows that, under assumptions such as preparation independence, distinct quantum states cannot correspond to overlapping distributions over the same underlying physical states. The theorem therefore rules out a broad class of ψ-epistemic models and supports a ψ-ontic reading of quantum mechanics. Bohmian mechanics already satisfies this criterion: the ontic state is given by the particle configuration *Q* together with the universal wave function Ψ, and distinct wave functions correspond to distinct physical states with different empirical consequences. This ψ-ontic status does not depend on whether Ψ is interpreted as an ontological field or as part of the dynamical structure in a nomological sense.

In the present framework, subsystem states are represented by the three–dimensional conditional wave functions $\psi_{i}\left(x,t\right)$ associated with particle positions $X_{i}\left(t\right)$. The proposal therefore preserves the ψ-ontic character of Bohmian mechanics while expressing the corresponding physical structure in terms of fields defined on ordinary three–dimensional space. Nothing in the PBR theorem fixes the space in which the quantum state is represented; it constrains the ontological status of the state, but not the space in which that state is represented.

Entanglement in this framework is not removed or redefined. It remains a property of the universal wave function, with standard measures of entanglement defined at the level of the configuration-space state. The present formulation instead provides a representation of how entangled correlations are realized dynamically in physical space. In particular, the dependence of each $\Phi_{i}\left(x,t\right)$ on the actual configuration of the remaining particles, together with the nonlocal coupling term $Q_{-i}\left(x,t\right)$ in Equation (6), encodes the dynamical influence of entanglement. In this way, correlations that are formally defined through the universal wave function are manifested through the coupled evolution of the three–dimensional fields. This perspective is consistent with standard treatments in Bohmian mechanics, where nonlocal correlations are encoded in the universal wave function while influencing particle dynamics through the guiding equation [4,7,8].

### 6.1. Nomological View

Dürr, Goldstein, and Zanghì have argued that the universal wave function Ψ in Bohmian mechanics is best understood as nomological, functioning as a global generator of particle motion rather than as a physical field in its own right [12]. A common objection to this view is that Ψ is explicitly time-dependent, whereas fundamental laws are often taken to be static. However, time dependence alone does not preclude a nomological role: in classical mechanics, a time-dependent Hamiltonian H(t) can encode a changing dynamical constraint without being regarded as a physical component of the system.

Our proposal adopts this nomological reading of the universal wave function but supplements it with additional ontological structure in ordinary three–dimensional space. While Ψ provides a compact, global encoding of the dynamics, the conditional wave functions associated with individual particles are taken to be ontic.

This nomological reading does not imply that Ψ is dispensable in specifying the mathematical state of the theory. Rather, it indicates that Ψ is not taken to represent an additional material field in physical space. The fact that Ψ must be specified to determine the evolution does not preclude this interpretation. In classical mechanics, the Hamiltonian must likewise be specified to generate the dynamics, and different physical systems correspond to different Hamiltonians. In that sense, the Hamiltonian is not part of the material ontology but part of the dynamical structure defining the system. The present proposal adopts an analogous stance: Ψ is required to fix the nonlocal coupling among the three–dimensional wave functions, but it is not thereby reinterpreted as a physical entity in space. Instead, it functions as part of the law-like structure governing the evolution of the ontological variables. In this respect, the universal wave function may be understood as specifying the dynamical structure of a given physical system rather than as part of its instantaneous material state.

### 6.2. Relation to Multi-Field Ontology

Recent work has proposed so-called “multi-field” interpretations, in which the universal wave function is reinterpreted as a single field on three–dimensional space whose values depend on tuples of spatial points [13]. While this does not treat configuration space as fundamental, it retains a single global object with inherently relational dependence and does not introduce distinct single-particle wave functions.

By contrast, the present framework associates with each particle a wave function defined on ordinary three–dimensional space, with an exact (though coupled) evolution equation derived from the universal dynamics. The resulting three–dimensional wave functions are particle-indexed fields that enter directly into the guiding equation. In this respect, the proposal differs from multi-field approaches not by eliminating the universal wave function but by organizing the ontology around distinct 3D fields defined on physical space while retaining Ψ within the dynamical structure.

### 6.3. Norsen’s Local Beables Program

Norsen’s local beables program [5] seeks to reconstruct quantum theory using entities defined exclusively on physical space, with the aim of avoiding commitment to a fundamental configuration-space ontology. The present proposal differs in strategy. It does not attempt to eliminate the universal wave function from the formalism. Rather, it retains Ψ within the standard Bohmian dynamics, adopting a nomological reading in the sense of Dürr, Goldstein, and Zanghì [12], while treating the three–dimensional wave functions derived from it as the ontological beables.

Equation (6) is obtained directly from the universal Schrödinger evolution together with the actual Bohmian configuration, so that the resulting dynamics remain exactly equivalent to standard Bohmian mechanics. In this sense, the proposal does not introduce new dynamical postulates but offers a reorganization of the ontology of the existing theory, expressing its dynamics equivalently in terms of coupled three–dimensional wave functions while preserving the full mathematical framework.

A further motivation for the present ontological reorganization concerns the relation between mathematical structure and ontological commitment. In physics, formalism alone does not uniquely determine what is taken to be physically real. The same Hamiltonians, dispersion relations, and field-theoretic structures can appear in different domains—for example in solid-state systems and high-energy models—while the underlying ontologies differ substantially. Mathematical equivalence therefore underdetermines questions about ontology: the formal structure constrains what is physically possible, but it does not by itself fix what entities are taken to exist (see, e.g., [6]).

Moreover, in Bohmian mechanics, all empirical interaction is realized in ordinary three–dimensional space. Particle trajectories evolve in that space, and the fields that guide them are naturally represented there as well. Detectors, measuring devices, and macroscopic records are likewise localized in ordinary three-dimensional space, where all empirical interaction takes place. While this fact does not logically rule out configuration-space realism, it suggests a natural methodological preference: when mathematically equivalent formulations are available, one may reasonably favor an interpretation in which the fundamental beables are defined in the same space in which dynamical processes and measurements occur.

For these reasons, the fact that the universal wave function Ψ is defined on configuration space does not by itself require configuration space to be treated as physically fundamental. The present proposal retains the standard Bohmian formalism while locating the ontological beables in ordinary three–dimensional space, where the particle dynamics are directly realized.

### 6.4. A Limited Analogy with Special Relativity

In a limited sense, our conceptual strategy bears comparison with Einstein’s 1905 reformulation of kinematics. In that case, the central mathematical structures of the theory—the Lorentz transformations—were already known from the work of Lorentz [14] and Poincaré [15], and Einstein’s contribution lay both in a conceptual reorganization and in providing a simpler derivation of the same equations from new physical principles [16].

The present work is more modest. We do not propose new principles or a new derivation of the fundamental equations. Rather, starting from the standard Bohmian formalism, we reorganize the ontology so that the theory is expressed in terms of entities defined in ordinary three–dimensional space, while remaining mathematically equivalent to the original description.

## 7. Conceptual Payoffs of the 3D Ontology

The statistical structure of the theory, including the recovery of Born’s rule via quantum equilibrium, was discussed in Section 4.1. The conceptual payoff of the present reorganization is structural rather than empirical. By treating particle-indexed wave functions defined on ordinary three–dimensional space as ontological beables, the dynamics of entangled systems may be described in terms of fields residing in physical space, while the universal wave function retains a nomological role within the dynamical framework.

This becomes particularly clear in measurement-like scenarios. In a two-slit experiment coupled to a which-way detector, the universal wave function is defined on the combined configuration space of particle and apparatus. In the present framework, however, each subsystem is associated with a three–dimensional wave function defined on ordinary space, allowing the entangled dynamics to be represented in terms of fields in physical space without altering the underlying Bohmian dynamics.

## 8. Classical Potentials and Holistic Dynamics

Before turning to a classical analogy, it is useful to recall the role of potentials in Bohmian mechanics. The guiding equation can be derived from a modified Hamilton–Jacobi formulation in which the quantum potential appears:

(8) $$Q\left(x_{1},\ldots ,x_{N},t\right)=-\sum_{i}\frac{\hbar^{2}}{2m_{i}}\frac{\nabla_{x_{i}}^{2}\left|\Psi \right|}{\left|\Psi \right|}.$$

This potential depends on the full configuration of the system and is therefore inherently nonlocal. It provides a concrete expression of how Bohmian dynamics encode holistic dependencies while remaining formulated in terms of variables associated with physical space.

A useful classical point of comparison is provided by vortex dynamics in incompressible fluids. For *N* point vortices in a two-dimensional ideal (inviscid, incompressible) fluid, the velocity of the *i*th vortex at position $r_{i}$ is given by the Biot–Savart law,

(9) $$\frac{dr_{i}}{dt}=\sum_{j\neq i}\frac{\Gamma_{j}}{2\pi }\frac{\hat{z}\times \left(r_{i}-r_{j}\right)}{|r_{i}-r_{j}{|}^{2}},$$

where $\Gamma_{j}$ denotes the circulation strength of the *j*th vortex and $\hat{z}$ is the unit vector perpendicular to the plane [17]. In this idealized incompressible setting, the motion of each vortex depends nonlocally, through a long-range kernel, on the positions of all others, yielding a holistic, non-factorizable dependence encoded entirely in ordinary physical space. In this case, no one infers from the nonlocal kernel that a higher-dimensional configuration space must be reified; the ontology remains fixed by the spatial variables that enter the equations of motion.

The instructive point of this analogy is not merely that holistic interactions can be formulated in real space but how ontology is naturally assigned within such a formulation. In vortex dynamics, the interaction kernel appearing in the equations of motion is not regarded as a physical entity; rather, the vortices themselves are taken to be the beables, even though their motion is determined by a collective interaction structure. The Biot–Savart term functions as a generator of motion, not as an object in the ontology.

The present framework invites a similar ontological reading. Although the dynamics of the 3D wave functions involve nonlocal coupling terms derived from the universal wave function, it is the particles and their associated three–dimensional wave fields that naturally present themselves as physical entities in space. The configuration-space wave function plays a role analogous to that of the interaction kernel in vortex dynamics: it encodes the structure of the coupling, but it is not itself the primary bearer of ontological commitment.

## 9. Conclusions

By elevating 3D wave functions to ontological primitives, we reinterpret Bohmian mechanics as a realist, dynamically nonlocal, yet spatially grounded theory. This shift reorganizes the ontology within ordinary three–dimensional space, where both particles and their guiding wave fields reside, without altering the empirical content or predictive structure of the theory. The contribution of the present work is not new dynamics or new predictions but a clarified ontological picture. By treating the universal wave function Ψ as nomological while assigning ontic status to particle positions and their associated 3D wave functions, the framework provides an alternative to configuration-space realism while remaining fully equivalent to standard Bohmian mechanics. What distinguishes this approach from earlier real-space proposals is that the 3D wave functions obey a precise evolution equation, Equation (6), derived directly from the universal Schrödinger dynamics together with the actual Bohmian configuration. This makes explicit that the dynamics of Bohmian mechanics can be organized in spatially grounded ontological terms, without supplementary postulates or modifications of the theory. Taken together, these results indicate the internal coherence and viability of an ontology based on particles and three–dimensional wave functions. The resulting formulation remains mathematically and empirically equivalent to the standard Bohmian framework.

## Data Availability

The original contributions presented in this study are included in the article material. Further inquiries can be directed to the corresponding author.

## Appendix A. Derivation of Equations (4)–(6)

For completeness, we outline how Equations (4)–(6) follow from standard Bohmian mechanics. The conditional wave function formalism and related derivations are discussed in detail in Refs. [4,5,7,8].

### Appendix A.1. Universal Dynamics

Let the universal wave function $\Psi (x_{1},\ldots ,x_{N},t)$ satisfy the *N*-particle Schrödinger equation

(A1) $$i\hbar \;\partial_{t}\Psi =\left[\sum_{k=1}^{N}\left(-\frac{\hbar^{2}}{2m_{k}}\nabla_{k}^{2}\right)+V\left(x_{1},\ldots ,x_{N}\right)\right]\Psi$$

and let the actual configuration $X_{k}\left(t\right)$ evolve according to the Bohmian guidance law

(A2) X˙k(t)=ℏmkIm∇kΨΨxj=Xj(t).

### Appendix A.2. Conditional (3D) Wave Function

For particle *i*, define the conditional (3D) wave function by conditioning on the actual positions of the remaining particles:

(A3) $$\psi_{i}\left(x,t\right):=\Psi \;\left(X_{1}\left(t\right),\ldots ,X_{i-1}\left(t\right),\;x,\;X_{i+1}\left(t\right),\ldots ,X_{N}\left(t\right),t\right).$$

This is the same object defined in Equation (3) of the main text.

### Appendix A.3. Time Derivative and Emergence of a Coupling Term

Differentiate (A3) using the chain rule:

(A4) $$\partial_{t}\psi_{i}={\left(\partial_{t}\Psi \right)}_{\mathrm{cond}}+\sum_{j\neq i}{\dot{X}}_{j}\left(t\right)\;{\left(\nabla_{j}\Psi \right)}_{\mathrm{cond}},$$

where “cond” denotes evaluation at $x_{j}=X_{j}\left(t\right)$ for all j≠i.

Substituting (A1) into the first term gives

(A5) $$i\hbar \;{\left(\partial_{t}\Psi \right)}_{\mathrm{cond}}=\left(-\frac{\hbar^{2}}{2m_{i}}\nabla_{x}^{2}+V_{i}\left(x,X_{-i}\left(t\right)\right)\right)\psi_{i}+\sum_{j\neq i}{\left(-\frac{\hbar^{2}}{2m_{j}}\nabla_{j}^{2}\Psi \right)}_{\mathrm{cond}},$$

where $V_{i}\left(x,X_{-i}\left(t\right)\right):=V\left(x_{1},\ldots ,x_{N}\right){|}_{x_{i}=x,\;x_{j\neq i}=X_{j}\left(t\right)}$ is the external/interparticle potential evaluated at the conditioned configuration.

Using the guidance Equation (A2) to rewrite the second term of (A4), and grouping all contributions involving derivatives with respect to the remaining coordinates (j≠i), one obtains an exact evolution equation of the form

(A6) $$i\hbar \;\partial_{t}\psi_{i}=\left(-\frac{\hbar^{2}}{2m_{i}}\nabla_{x}^{2}+V_{i}\left(x,X_{-i}\left(t\right)\right)\right)\psi_{i}+C_{i}\left(x,t\right),$$

where $C_{i}\left(x,t\right)$ collects all “environment” contributions generated by the universal dynamics when conditioning on $X_{-i}\left(t\right)$.

### Appendix A.4. From C i to the Main Text Structure

Equation (A6) is equivalent to Equation (4) in the main text, i.e., $C_{i}\left(x,t\right)=K_{i}\left(x,t\right)\;\psi_{i}\left(x,t\right).$ To make the structure of $K_{i}$ explicit, one writes the universal wave function in polar form $\Psi =Re^{iS/\hbar }$ and uses the guidance law (A2). Standard manipulations (see Refs. [4,7,8]) reorganize $K_{i}$ into the separated form reported in Equation (5) of the main text: a position-dependent contribution $Q_{-i}\left(x,t\right)$ (built from the quantum potentials of the other degrees of freedom, evaluated at the actual configuration) plus terms depending only on time.

Since purely time-dependent terms correspond to spatially uniform phases, they can be removed by a gauge transformation $\psi_{i}\left(x,t\right)=e^{\Lambda (t)}\Phi_{i}\left(x,t\right)$ without changing the guidance equation. This yields the final evolution equation for the rescaled 3D wave function $\Phi_{i}$, namely, Equation (6) of the main text:

$$i\hbar \frac{\partial \Phi_{i}}{\partial t}=\left(-\frac{\hbar^{2}}{2m_{i}}\nabla_{x}^{2}+V_{i}\left(x,X_{-i}\left(t\right)\right)\right)\Phi_{i}+Q_{-i}\left(x,t\right)\;\Phi_{i}.$$

Thus, Equations (4)–(6) follow directly from the universal Schrödinger Equation (A1), together with the Bohmian guidance law (A2).
